# Extended regions of suspected mis-assembly in the rat reference genome

**DOI:** 10.1038/s41597-019-0041-6

**Published:** 2019-04-23

**Authors:** Shweta Ramdas, Ayse Bilge Ozel, Mary K. Treutelaar, Katie Holl, Myrna Mandel, Leah C. Solberg Woods, Jun Z. Li

**Affiliations:** 1 Program in Bioinformatics, University of Michigan, Ann Arbor, Michigan USA; 2 Department of Human Genetics, University of Michigan, Ann Arbor, Michigan USA; 3 Department of Internal Medicine, University of Michigan, Ann Arbor, Michigan USA; 4 Department of Pediatrics, Human and Molecular Genetics Center and Children’s Research Institute, Medical College of Wisconsin, Milwaukee, Wisconsin USA; 5 National Institutes of Health, Bethesda, Maryland USA; 6 Department of Internal Medicine, Wake Forest School of Medicine, Winston-Salem, North Carolina USA; 7 Department of Computational Medicine and Bioinformatics, University of Michigan, Ann Arbor, Michigan USA

**Keywords:** Sequence annotation, Genome informatics, Rat, Genome

## Abstract

We performed whole-genome sequencing for eight inbred rat strains commonly used in genetic mapping studies. They are the founders of the NIH heterogeneous stock (HS) outbred colony. We provide their sequences and variant calls to the rat genomics community. When analyzing the variant calls we identified regions with unusually high levels of heterozygosity. These regions are consistent across the eight inbred strains, including Brown Norway, which is the basis of the rat reference genome. These regions show higher read depths than other regions in the genome and contain higher rates of apparent tri-allelic variant sites. The evidence suggests that these regions may correspond to duplicated segments that were incorrectly overlaid as a single segment in the reference genome. We provide masks for these regions of suspected mis-assembly as a resource for the community to flag potentially false interpretations of mapping or functional results.

## Introduction

The laboratory rat (*Rattus norvegicus*) is an important model organism for studying the genetic and functional basis of physiological traits. Compared to the mouse, the rat shows a greater similarity to humans in many complex traits^1^ and has been widely used in physiological, behavioral and pharmacological research. With the arrival of high-throughput genotyping and sequencing technologies, the rat has also been used in genetic studies to map causal loci, or identify genes that affect disease-related traits.

An essential resource in such genetic studies is the rat reference genome^2^, which provides the coordinate system to orderly manage our rapidly increasing knowledge of rat genes, their regulatory elements, gene products and variants, functional profiles of diverse tissues, as well as biological dysregulation in disease models. The reference genome is also the basic map in comparative analyses that focus on the evolutionary relationship among rat strains or between the rat and other organisms.

Gene discovery studies using animal models can be roughly classified by the type of mapping populations adopted. Currently, popular genetic systems involving the rat include naturally occurring outbred populations, laboratory-maintained diversity outbred populations, inbred line-based crosses (e.g., F2-crosses, or advance inbred lines), recombinant inbred lines, and many others. Regardless of the system, a comprehensive knowledge of DNA variation in the mapping population is essential for both the study design and biological interpretation. In this study, we sought to use whole-genome sequencing (WGS) to ascertain DNA variants in eight inbred strains: ACI, BN, BUF, F344, M520, MR, WKY and WN, which are founders of the NIH Heterogeneous Stock (HS) population. The HS rat has been used in genetic studies of metabolic and behavioral traits^3–8^. WGS data for these eight strains have been previously described^9,10^, using the SOLiD technology. Here we present WGS results from the Illumina technology, containing genotypes at ~16.4 million single-nucleotide variant (SNV) sites. We expect that the sequences of the eight HS founders and fully-ascertained DNA variations can aid the imputation, haplotyping, and fine mapping efforts by the rat genomics community.

When analyzing the SNV data we noted that, while the eight founders are inbred, all contain an unusually high amount of heterozygous nucleotide positions. Remarkably, these sites tend to concentrate in hundreds of discrete genomic regions, which collectively span 6–9% of the genome. We show that the heterozygous genotypes tend to recur in multiple, if not all, of the eight strains, and that the suspected regions tend to have higher-than-average read depths. We propose that these regions can be explained by mis-assembly of the rat reference genome, where many of the highly repetitive segments may exist in tandem or dispersed in distant regions, but have been erroneously “folded” in the current coordinate system, causing the reads of high homology that originate in distinct homozygotic regions to falsely aggregate to produce apparent heterozygous calls. This interpretation is not unexpected when one compares the genome assembly statistics between mouse and rat: the latest release of the mouse reference genome, GRCm38.p6, contains 885 contigs and a contig N50 length of 32.3 megabases; whereas the rat reference genome, rn6, has 75,687 contigs and N50 of 100.5 kilobases. With this report we release mask files for the suspected regions, so that they can be used to flag questionable results in current genomic studies until the time when a revised, more accurate reference assembly becomes available.

## Results

### Description of the variant calls

DNA from one female animal for each of the eight strains was sequenced^11^ (Methods). Median read depth over the genome ranges 24X–28X across the eight samples. Joint variant calling revealed 16,405,184 post-filter single-nucleotide variant sites^12^ on the autosomes and chromosome X. The number of heterozygous sites per strain varies from 1,560,708 (BN) to 2,160,032 (M520) (Supplementary Table S1). BN represents the reference genome, and has more Ref/Ref than Alt/Alt genotypes. In contrast, the other seven strains have a comparable number of Ref/Ref calls as Alt/Alt calls.

We compared our genotype calls with those from Hermsen *et al*.^10^ by calculating between-study concordance rates at sites reported in both, and using genotypes that do not include the missing calls. Supplementary Table S2 shows that each of the eight lines can be correctly matched between the two datasets, confirming the sample identity even when the two studies were based on different animals for a given line. BN has the highest between-study concordance: 0.95. Six other lines have concordance >0.86. However, MR has the lowest concordance, 0.69. To determine if an animal from another line was mislabeled as MR, we compared our MR data with a larger panel of 42 strains previously published^10^ and found that our MR had the highest match with MR and WAG-Rij in that study. This suggests that the MR lines in different laboratories may have diverged to an unusually large degree.

### Regions of unexpected high-heterozygosity

**Fig. 1:**
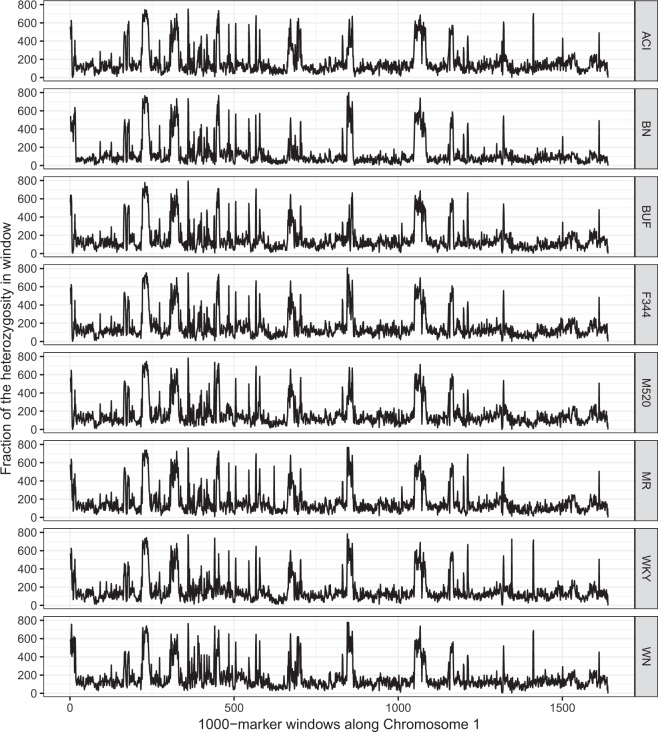
Consistent heterozygosity patterns across the eight lines. Shown are the fractions of heterozygous genotypes (y-axis) in non-overlapping 1000-SNV windows in chromosome 1, displayed for the eight inbred lines.

The eight founder strains were kept as inbred lines over many generations, thus were expected to show low heterozygosity across the genome^13^. However, when we calculated the fraction of heterozygous genotypes in consecutive 1000-SNV windows, we observed highly varied distribution of this metric along the genome. Not only were there many windows of high heterozygosity (>0.25), they also tended to recur in multiple lines (Fig. 1). Some of these windows were found in all 8 strains (Supplementary Fig. S1), including BN, the strain of the reference genome.

We chose a heterozygosity cutoff value of 25% based on the distribution of per-window heterozygosity (Supplementary Fig. S2). After merging nearby high-heterozygosity (abbreviated as “high-het”) windows if they are separated by a single low-het window with heterozygosity rate >0.175, we obtained 304-482 contiguous high-het regions across the eight lines (median 452), covering 176.4–254.8 Mb, equivalent to 6.3–9.2% of the genome (average 8.4%). The distribution of segment lengths is shown in Supplementary Fig. S3. Of the individual heterozygous calls in each line, 28–31% fall in the high-het regions (mean 29%). Of the 534,266 triallelic variant sites, 484,583 (~91%) fall in the regions that are high-het in at least one strains, covering 12% of the genome.

The high-heterozygous regions found in all eight lines contain 1,756 Ensembl genes. These genes were enriched for G-protein coupled receptors and olfactory receptors (the enrichment for these gene sets were 3.2- and 2.0-fold respectively, Benjamini-Hochberg FDR <0.01), which are known to have many paralogous copies in mammalian genomes^14^. There are 4,963 missense variants, 123 stop-gain variants, and 154 splice donor/acceptor variants in 372 genes in these regions. In the future, more biological lessons could be learned regarding how some highly “fluid” gene families may or may not co-localize to regions prone to rapid expansion, perhaps mediated by, or resulting in, segmental duplications.

### Heterozygous calls tend to be recurrent and show higher read depths

**Fig. 2:**
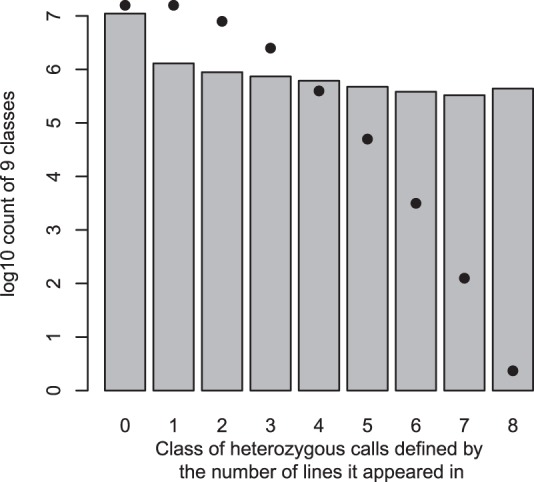
Recurrence pattern of heterozygous genotypes across 8 founders. All variant sites were categorized as having heterozygous genotypes in 0, 1,…, up to 8 lines. The bar graph displays the observed site counts in the 9 categories, while the dots show the expected number of sites if recurrence is random, estimated under a simple binomial model.

While Supplementary Fig. S3 shows that 1000-variant windows of high heterozygosity tend to appear in multiple lines, we also analyzed individual heterozygous genotypes to see how much they tend to recur in multiple lines. We divided the ~16.4M variant sites based on the number of heterozygous genotypes observed in the eight lines, thus defining nine variant site categories, for a site being heterozygous in 0 to 8 lines (Fig. 2). If the heterozygous genotypes appear independently in the eight lines with a probability of p, the expected chance of seeing a site with two heterozygotes (that is, in two of the eight lines) would be proportional to p^2^, and in three lines: p^3^. We estimated the upper limit of p by counting the fraction of heterogeneous genotypes over all 8 lines in all sites, knowing that this fraction is already biased upward due to the highly recurrent heterozygous sites. The expected probability of seeing k heterozygous sites, a(k)p^k^, where a(k) is the coefficient of sampling k out of 8 in the binomial distribution, drops much faster than the observed k-het counts (Fig. 2). For instance, the observed number of sites that are heterozygous in all eight lines is more than five orders of magnitude higher than expectation.

**Fig. 3:**
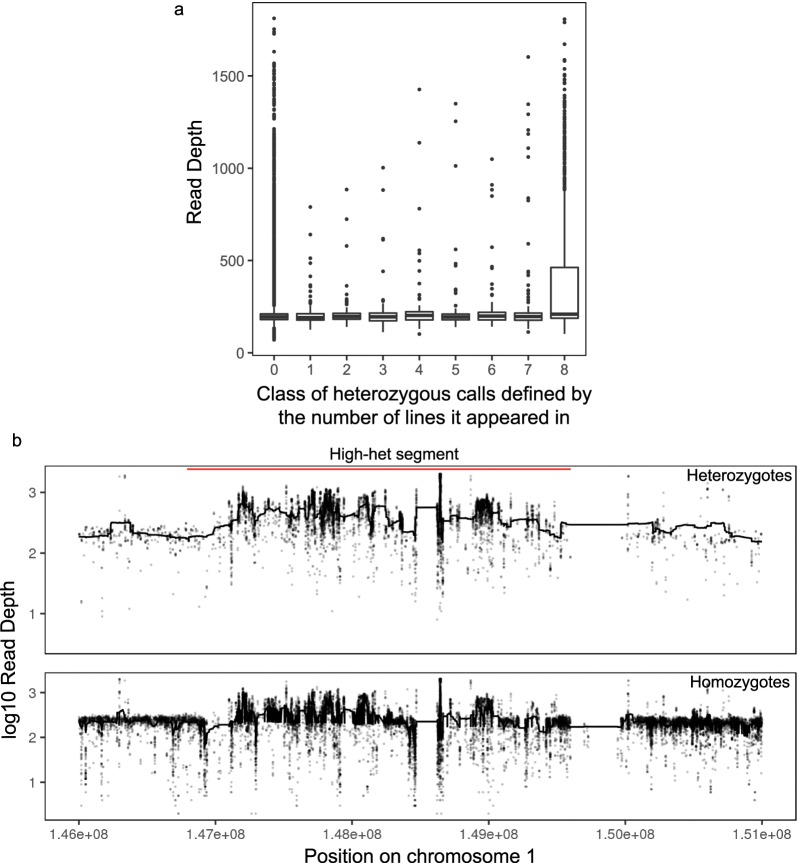
Higher read depths in high-heterozygosity windows. (**a**) Boxplot of per-window average read depth stratified on the x-axis by the number of lines for which a given window is classified as high-het. It shows that windows with high-het in all eight lines tend to show higher average read depth. (**b**) An example of a 5 Mb region in Chromosome 1 and line AC, with a high-het region in the middle. Y axis is the read depth for individual sites, showing that in the high-het window, both the heterozygosity calls (upper panel) and homozygosity calls (lower panel) show higher read depth and often higher variance of read depth.

The tendency for an individual site to appear heterozygous in multiple lines is related to higher read depth at these sites. Figure 3a shows that windows that are high-het in all 8 lines tend to have higher average read depths than other windows. Figure 3b shows an example where low-het regions tend to have lower, and more stable, read depth than nearby regions of high heterozygosity. Notably, the read depth is higher in high-het regions for both homozygous and heterozygous genotypes.

### Concordance with results from a different platform

We analyzed the previously released variant calls from Hermsen *et al*. 2015 (after converting the coordinates of these calls to the rn6 genome build from the original rn5) to see if our high-heterozygosity segments were also found in their calls. The Hermsen dataset revealed 5-18% (median 14%) of the genome in high-het windows. We found that these high-het regions tend to match in the two call sets (an example in Supplementary Fig. S4), with an overlap of 24–36% as defined by the length of the intersect of segments called high-heterozygosity in both calls, divided by the union of regions defined as high-heterozygosity in either call.

We have compiled a list of these high-het regions of the genome and provided them as bed files on our GitHub archive^15^ (under the folder “merged”).

## Discussion

In this study we created WGS-based variant call sets for eight inbred lines which represent the genomic source of the multi-parental HS population. Our data suggest that the current rat reference genome contains hundreds of problematic regions where inbred lines show increased apparent heterozygosity and higher-than-usual read depth. These regions make up ~8.4% of the genome, and likely represent regions of mis-assembly. In other words, a properly unfolded genome may be ~8% longer.

Several lines of evidence led us to the interpretation that regions of high-heterozygosity likely represent an artifact where two or more segments of high similarity are incorrectly “collapsed”, or “folded”, into the same segment in the rat reference genome. First, the rate of heterozygous calls is higher than what one would expect for inbred lines; and these heterozygous calls cluster in discrete regions. Second, these high-heterozygosity regions tend to recur, sometimes in all eight lines, indicating that they are unlikely to have arisen from genetic drift or strain-specific selection. Finally, these regions tend to have higher read depth than surrounding regions. We do not think these are regions of copy number variation, because many appear in all 8 strains analyzed, not a strain-specific deviation from the reference. In other words, while copy number variation might explain some of the high-het regions, a more plausible explanation for most of such regions is that the reference needs to be revised. The current data, however, do not rule out the possibility that some suspected regions may contain some genes or gene families with genuinely high levels of polymorphism, or genuinely high degrees of paralogous expansion. The rat reference genome, unlike the human and the mouse reference genomes, was assembled using a hybrid of shotgun-sequencing and clone-based approaches. Our results suggest that this mixed (primarily shotgun) approach has not been able to resolve all the repetitive regions. Thus, Illumina sequence reads that originated in distinct but highly similar regions–some of these may fit the strict definition of paralogous regions–are incorrectly aligned to the same collapsed region in the reference genome, producing false heterozygous calls.

Guryev *et al*.^16^ studied the rat reference genome (rn4 at the time) and used the read-depth distribution to identify 73 regions of suspected mis-assembly, which make up ~1% of the genome. Only 2 of these 73 regions can be lifted over to the rn6 genome and both were called high-het in our data. However, they didn’t analyze the distribution of heterozygous genotypes. Another study performed WGS on the inbred strain SHR/Olalpcv and observed higher-than-expected levels of heterozygosity. The authors suggested that this could have resulted from collapsing of reads from segmental duplications^17^, an interpretation similar to ours. Because they analyzed a single strain, the data did not show the consistency of suspected regions across strains.

In an early draft genome of the mouse, segmental duplications were inadvertently misassembled^18^. Our estimates of misassembled regions underscore a similar problem with the current reference genome for the rat. These regions harbor more than 1,700 genes, with 5,000 apparent missense variants that may be an artifact. Genomic studies that fail to flag these regions are at risk of reporting incorrect candidates for downstream studies. We propose that the community apply the mask files provided here^15^, until a more refined reference genome becomes available.

## Methods

### Animals, DNA samples, whole-genome sequencing

Eight animals, one for each of the eight founders of the HS population, were used in the study. Tissue samples of the original founder strains was obtained from NIH (M.M.). DNA was extracted at the University of Michigan (Ann Arbor, MI) from the liver of seven animals (ACI/N, BUF/N, F344/N, M520/N, MR/N, WKY/N, WN/N), and from the tail of a BN/N animal. All animals were female. The DNeasy Blood and Tissue Kit from Qiagen (Hilden, Germany) was used for DNA extraction. Samples were further QC’d and sequenced at *Novogene* (Beijing, China) following the standard Illumina protocols. Library preparation produced fragment libraries of ~350 bp insert length. Sequencing was done on Illumina HiSeqX-Ten to collect 150 bp paired-end data, aiming for an average depth of 25X per strain. We applied Illumina sequencing in this study as it is compatible with most of the existing data. The comparison with the SOLiD data is useful as it examined the concordance between our results and the previously largest re-sequencing dataset for the rat.

### Sequence alignment and variant calling

We aligned the raw sequence reads to the rat reference genome (rn6) using *BWA* version 0.5.9^19^, removed duplicates using *Picard* v1.76, and performed realignment, recalibration and joint variant calling across eight strains with the UnifiedGenotyper with *GATK* v3.4^20^. We removed variant sites with fewer than 10 reads in eight samples, and variant site quality score (QUAL) <=30. We chose not to use the HaplotypeCaller as we have only eight inbred lines, which are not the population-based samples suitable for building haplotypes.

Chromosome X data showed the same pattern of heterozygosity as the autosomes in all eight animals, thus confirming that they are female. We excluded the Y chromosome calls in downstream analysis. We did not call indels in this data release.

For comparison purposes we also ran the analysis with (1) two earlier versions of the reference genome, rn4 and rn5; (2) two other aligners. The first is by feeding the *BWA* aligned files into *Stampy*^21^ version 1.0.32. *Stampy* alignment shows higher sensitivity than BWA, especially when reads include sequence variation^21^. (The use of BWA-alignment as input for Stampy is to increases alignment speed without reducing sensitivity). The second is *Bowtie2* v2.1.0^22^. All the post-alignment processes followed the same *Picard* and *GATK* steps. The results show high concordance among the genome versions (Supplementary Table S3) and among the aligners (Supplementary Tables S4, S5).

### Comparison with the previously published variant calls using SOLiD

We compared our variant call set (for *BWA* alignment and rn6) with that by Hermsen *et al*.^10^, which was based on the SOLiD sequencing data. As that call set was aligned to rn5, we lifted over the variants to the rn6. We used the *Liftover* tool provided by the UCSC genome browser. *Liftover* converts genome coordinates and genome annotation files between assemblies.

### Defining regions of unusually high rates of heterozygosity

The final call set contains >16.4M SNV sites. We divided the genome into 1000-site windows, with a median window length of 221,100 bases. For each of the eight samples and in each window we computed the fraction of heterozygous sites (only using the number of non-missing sites in that window as the denominator). Based on the inflection point in the empirical distribution of this per-window heterozygosity fraction (Supplementary Fig. S2) we chose a cutoff of 25% to designate windows as of high-heterozygosity. We concatenated adjacent windows of high-heterozygosity into the same segment, and in a second step, merged adjacent high-het segments if they are separated by a single “low-heterozygosity” window, if that window had more than 0.175 heterozygote rate (Supplementary Fig. S5). After merging, there is no evidence of many very short low-het segments separating high-het segments (Supplementary Fig. S3).

### Gene set enrichment analysis

We first obtained the set of genes in the high-het regions using the UCSC table browser. We then performed pathway enrichment on these genes using *DAVID* v6.8^23^ and analyzed results from the functional annotation clustering tool, using the rat reference genome as the background set. The enrichment ratio was calculated based on the 2-by-2 contingency table, tabulating the numbers of all genes (n1) and those in high-het regions (n2) and, for a given gene set, those in the set (n3) and those in the high-het regions (n4). We then obtain an enrichment ratio of n4*n1/(n2*n3).

## Supplementary Information

Supplementary Materials

## Data Availability

The datasets generated during and/or analyzed during the current study are available in the NCBI Sequence Read Archive repository^11^ and *Figshare*^12^.

## Code Availability

Our GitHub archive^15^ contains the scripts used in the sequence analysis (file “sequencingscripts.sh” in the folder “scripts”) and in downstream analysis and figure plotting (under the folder “scripts”).
