# Supplementary material for: Extended regions of suspected mis-assembly in the rat reference genome
Source: Sci Data. 2019 Apr 23;6:39. doi: 10.1038/s41597-019-0041-6 (PMC6478900; doi:10.1038/s41597-019-0041-6)

Supplemental Materials for "Extended regions of suspected mis-assembly in the rat reference genome"

Table of Content:

Supplementary Table S1. **p.2**

Genotype counts and heterozygote frequencies in the eight lines.

Supplementary Table S2. **p.3**

Concordance rate between our variant calls and the previous variant calls from Hermsen et al, 2015.

Supplementary Table S3. **p.4**

Consistence across the three recent versions of the rat reference genome.

Supplementary Table S4. **p.5**

Consistency of high-heterozygosity regions among three alignment methods.

Supplementary Table S5. **p.6**

High levels of genotype concordance among variant calls obtained from different aligners (*BWA*, *Bowtie*, *BWA-Stampy*) followed by calling with UnifiedGenotyper.

Supplementary Figure S1. **p.7**

Histogram of the number of 1000-SNV windows that are high-het (heterozygosity > 0.25) in, from left to right, 0, 1, …, 8 founder lines.

Supplementary Figure S2. **p.8**

Distribution of the heterozygosity level in 1000-SNV windows for each of the eight lines, suggesting that 0.25 is a reasonable cutoff for defining high-het windows.

Supplementary Figure S3. **p.9**

Distribution of high-het segment lengths in the 8 lines.

Supplementary Figure S4. **p.10**

Similar regional patterns of high-het segments in Chr2 between this study and the previously reported SOLiD calls.

Supplementary Figure S5. **p.11**

Distribution of heterozygosity rates in windows that are initially classified as low-het, but are flanked by high-het windows on both sides.

Supplementary Table S1. Genotype counts and heterozygote frequencies in the eight lines. BN represents the reference genome and shows an outlier pattern for most metrics. 0/0, 0/1, 1/1 refers to Ref/Ref, Ref/Alt, and Alt/Alt genotypes. ./. refers to no-call due to low genotype quality.

Strain	0/0	0/1	1/1	./.	Het frequencies	Sites with > 2 alleles
ACI	6,249,928	2,096,993	5,421,143	2,385,991	0.150	251,129
BN	12,324,354	1,560,708	803,800	1,623,744	0.106	92,578
BUF	6,688,095	2,058,214	5,192,072	2,218,374	0.147	248,429
F344	6,587,382	2,101,058	5,282,082	2,181,114	0.150	253,548
M520	6,628,579	2,160,032	5,329,841	2,031,084	0.150	255,648
MR	6,736,380	2,111,216	5,243,452	2,063,655	0.149	250,481
WKY	6,599,091	1,976,801	5,130,247	2,456,145	0.144	242,900
WN	5,993,693	2,114,990	5,734,351	2,304,637	0.150	257,513

Supplementary Table S2. Concordance rate between our variant calls and the previous variant calls from Hermsen et al, 2015. The 8-by-8 table contains concordance values for all pair of samples. Here, we define concordance as the number of variant sites with the same genotype calls in both call sets divided by the number of variant sites with non-missing calls in both call sets.

		**Hermsen *et al*. 2015 calls**							
		**ACI**	**BN**	**BUF**	**F344**	**M520**	**MR**	**WKY**	**WN**
**Our variant calls**	**ACI**	0.874	0.478	0.577	0.588	0.584	0.599	0.514	0.547
	**BN**	0.52	0.952	0.548	0.541	0.55	0.559	0.497	0.551
	**BUF**	0.586	0.511	0.871	0.628	0.631	0.609	0.520	0.599
	**F344**	0.594	0.501	0.625	0.873	0.674	0.593	0.520	0.594
	**M520**	0.591	0.511	0.63	0.676	0.873	0.601	0.514	0.592
	**MR**	0.585	0.521	0.605	0.599	0.598	0.685	0.526	0.603
	**WKY**	0.518	0.453	0.516	0.518	0.51	0.536	0.859	0.508
	**WN**	0.55	0.511	0.594	0.591	0.588	0.599	0.508	0.88

Supplementary Table S3. Consistence of variant calls using the three recent versions of the rat reference genome. The tables show the cross-tabulation of the number of genotype calls (in unit of 1,000) between rn4 and rn6 (upper table), and between rn5 versus rn6 (lower). The overall concordance is 0.98 between rn5 and rn6, and 0.97 between rn4 and rn6.

		**rn6**			
		**0/0**	**0/1**	**1/1**	**./.**
**rn4**	**0/0**	37,196.7	594	140.1	152
	**0/1**	48	839	133.3	10.6
	**1/1**	5.9	303.2	24958.4	155.1
	**./.**	537.8	498.6	1112.4	1920.8

	**rn6**				
		**0/0**	**0/1**	**1/1**	**./.**
**rn5**	**0/0**	13908.1	374.7	59.8	240.6
	**0/1**	48.2	1410.5	74.6	19.7
	**1/1**	2.5	170.9	9606.8	351.7
	**./.**	72.3	65.6	103.3	364

Supplementary Table S4. Consistency of high-heterozygosity regions across variant calls using three alignment methods. Shown are the total length of high-het regions for the 8 lines and with the use of the three aligners: *BWA*, *Bowtie2* and *BWA-Stampy*.

As the background level of heterozygosity differ among the three call sets, we applied different het fraction thresholds to define high-het windows: 0.25 for *BWA*, 0.175 for *Bowtie2*, and 0.2 for *Stampy*. The last three columns list concordance rates between heterozygous segments called by a pair of aligners, defined as intersect/union of the segments.

	BWA	***Bowtie***	***Stampy***	***BWA:Bowtie***	***BWA: Stampy***	***Bowtie: Stampy***
**ACI**	238,523,232	260,711,962	302,820,837	0.570	0.540	0.510
**BN**	171,689,865	189,496,386	213,984,578	0.610	0.600	0.580
**BUF**	223,339,767	247,060,856	289,737,173	0.550	0.550	0.490
**F344**	236,379,041	252,011,886	293,848,725	0.560	0.540	0.510
**M520**	243,992,401	243,643,168	294,212,048	0.580	0.540	0.500
**MR**	225,952,435	263,629,202	276,523,184	0.550	0.550	0.500
**WKY**	242,849,594	239,016,453	288,897,012	0.570	0.540	0.520
**WN**	228,435,487	249,395,735	298,824,484	0.530	0.520	0.470

Supplementary Table S5. High levels of genotype concordance among variant calls obtained from different aligners (*BWA*, *Bowtie*, *BWA-Stampy*) followed by calling with UnifiedGenotyper. As before, we define concordance as the number of variant sites with the same genotype calls in both call sets divided by the number of variant sites with non-missing calls in both call sets.

	BWA	***Bowtie***	***Stampy***
BWA	1	0.972	0.972
Bowtie		1	0.974
Stampy			1

Supplementary Figure S1. Histogram of the number of 1000-SNV windows that are high-het (heterozygosity > 0.25) in, from left to right, 0, 1, …, 8 founder lines.

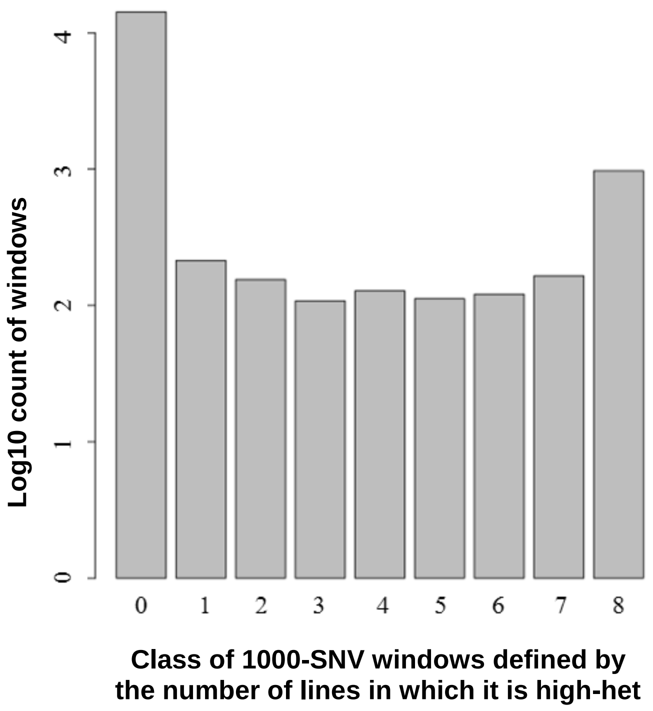

Supplementary Figure S2. Distribution of the heterozygosity level in 1000-SNV windows for each of the eight lines, showing that 0.25 is at the "elbow" and is a reasonable cutoff for defining high-het windows.

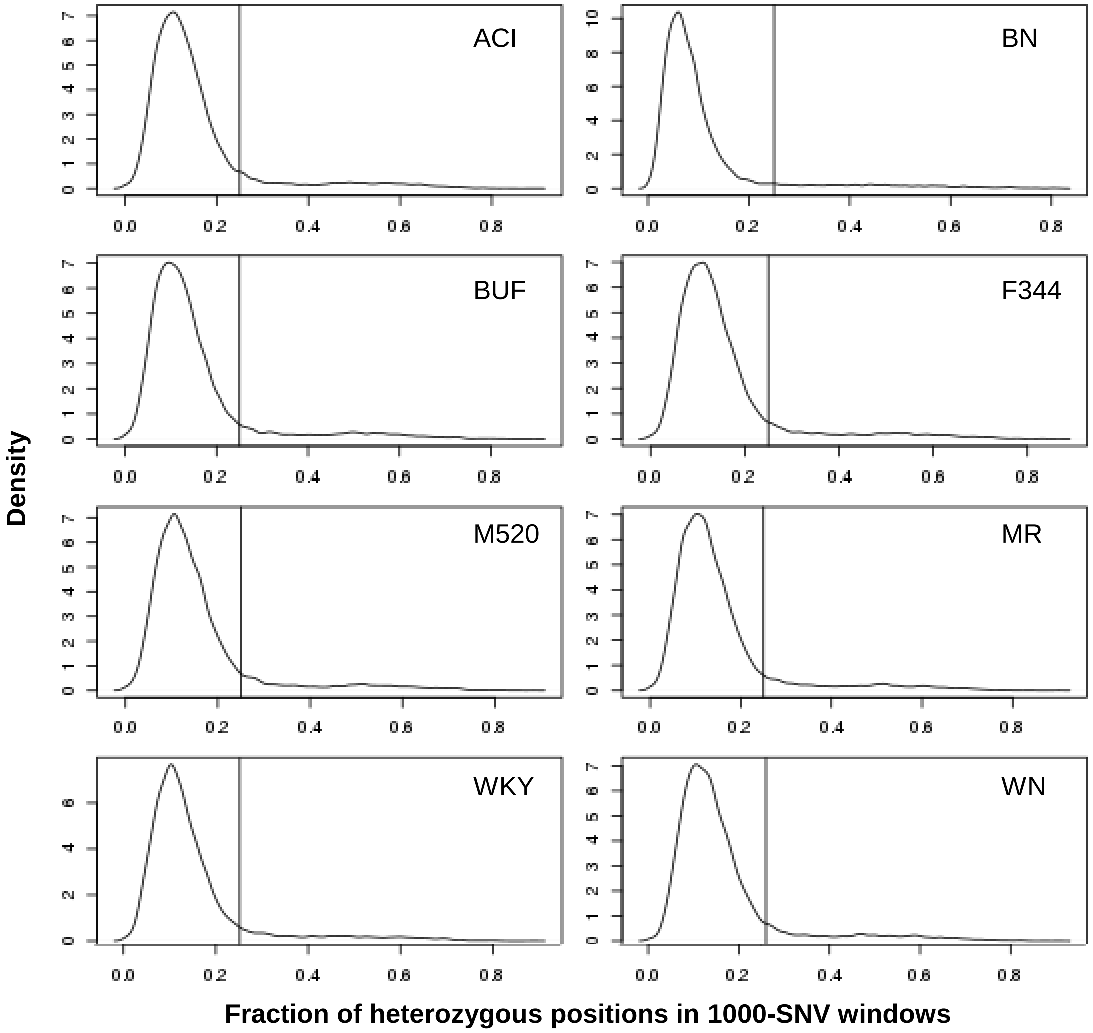

Supplementary Figure S3. Distribution of high-het segment lengths in the 8 lines. Here x=6.0 corresponds to 1 Mb.

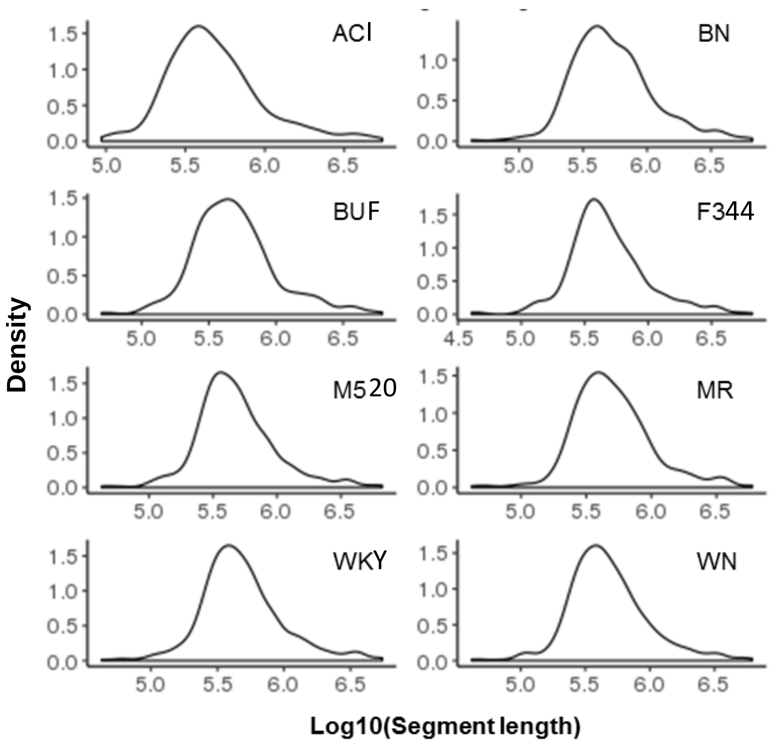

Supplementary Figure S4. Similar regional patterns of high-het segments in Chr2 between this study and the previously reported SOLiD calls. The x-axis is displayed as base positions of the 1000-SNV windows rather than the window IDs, because the two call sets (Illumina and SOLiD) contained different numbers of variant sites, and the positions of the 1000-SNV windows are mismatched between the two. The horizontal line represents 100 heterozygous sites in a 1,000-SNV window.

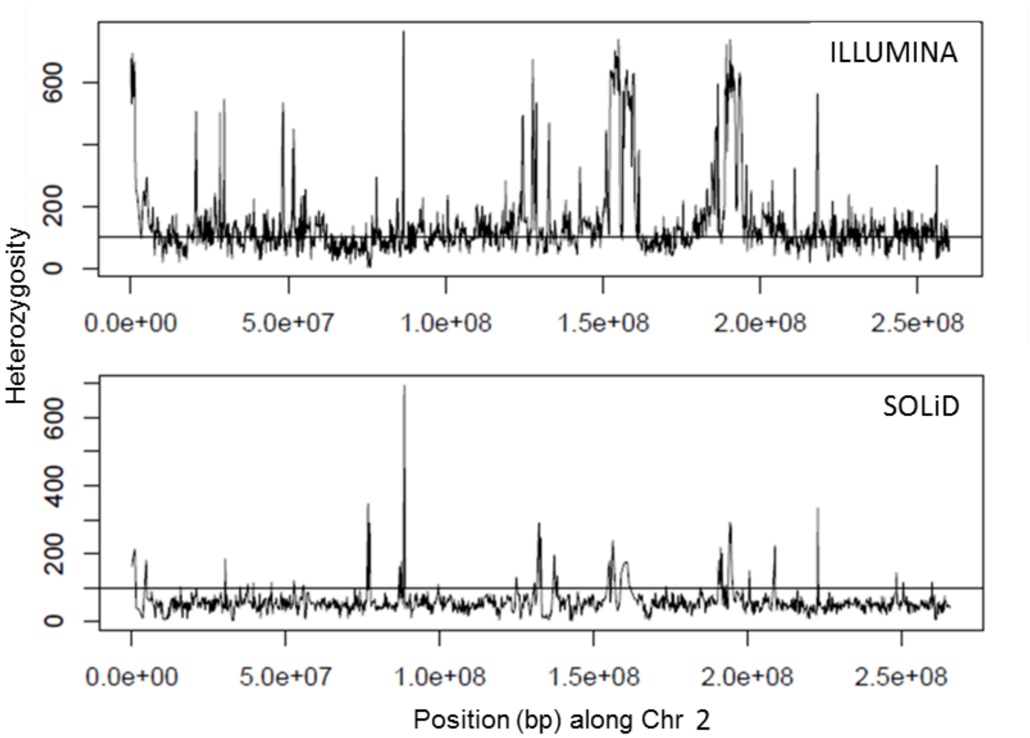

Supplementary Figure 5. Distribution of heterozygosity rates in windows that are initially classified as low-het, but are flanked by high-het windows on both sides. The bimodal distribution suggests those with >175 hets may be merged with flanking high-het segments.

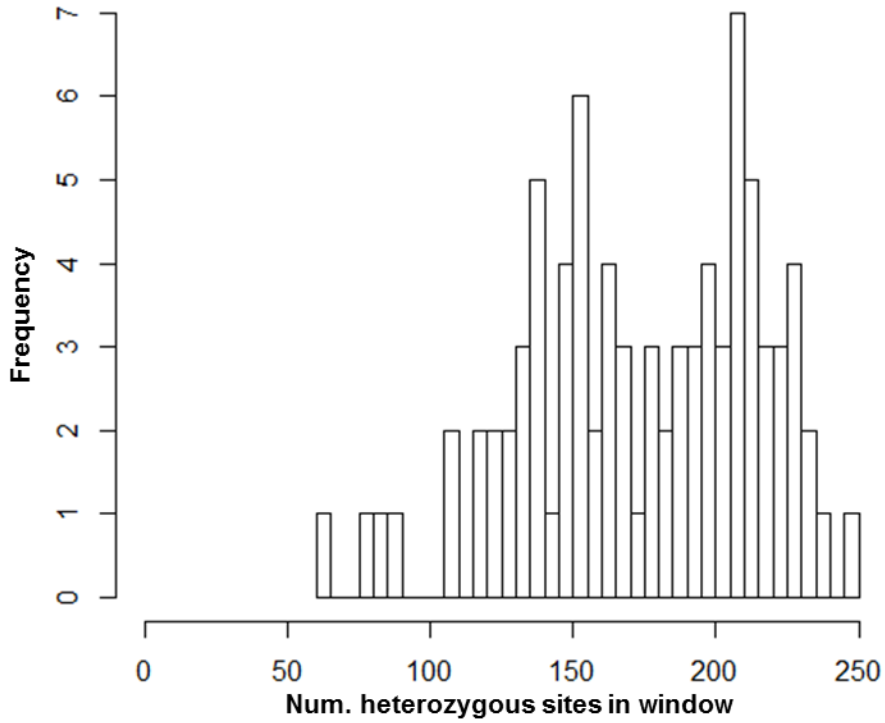

Supplement: Supplementary file 1 — Supplementary Materials [file 41597_2019_41_MOESM1_ESM.pdf]
